# Towards a validated musculoskeletal knee model to estimate tibiofemoral kinematics and ligament strains: comparison of different anterolateral augmentation procedures combined with isolated ACL reconstructions

**DOI:** 10.1186/s12938-023-01094-y

**Published:** 2023-03-27

**Authors:** Sara Sadat Farshidfar, Joseph Cadman, Thomas Neri, David Parker, Richard Appleyard, Danè Dabirrahmani

**Affiliations:** 1 Macquarie Medical School, Faculty of Medicine, Health and Human Sciences, Macquarie University, Sydney, NSW Australia; 2 Sydney Orthopaedic Research Institute, Sydney, Australia; 3 Department of Orthopaedic Surgery, University Hospital of Saint Etienne, Saint Etienne, France; 4 EA 7424-Inter-University Laboratory of Human Movement Science, University Lyon-University Jean Monnet Saint Etienne, Saint Etienne, France

**Keywords:** Musculoskeletal knee model, ACL reconstruction, Lateral augmentation, Anterolateral ligament, Ligament loading, Ligament strain, Forward dynamics, Inverse kinematics, Model validation, OpenSim

## Abstract

**Background:**

Isolated ACL reconstructions (ACLR) demonstrate limitations in restoring native knee kinematics. This study investigates the knee mechanics of ACLR plus various anterolateral augmentations using a patient-specific musculoskeletal knee model.

**Materials and methods:**

A patient-specific knee model was developed in OpenSim using contact surfaces and ligament details derived from MRI and CT data. The contact geometry and ligament parameters were varied until the predicted knee angles for intact and ACL-sectioned models were validated against cadaveric test data for that same specimen. Musculoskeletal models of the ACLR combined with various anterolateral augmentations were then simulated. Knee angles were compared between these reconstruction models to determine which technique best matched the intact kinematics. Also, ligament strains calculated by the validated knee model were compared to those of the OpenSim model driven by experimental data. The accuracy of the results was assessed by calculating the normalised RMS error (NRMSE); an NRMSE < 30% was considered acceptable.

**Results:**

All rotations and translations predicted by the knee model were acceptable when compared to the cadaveric data (NRMSE < 30%), except for the anterior/posterior translation (NRMSE > 60%). Similar errors were observed between ACL strain results (NRMSE > 60%). Other ligament comparisons were acceptable. All ACLR plus anterolateral augmentation models restored kinematics toward the intact state, with ACLR plus anterolateral ligament reconstruction (ACLR + ALLR) achieving the best match and the greatest strain reduction in ACL, PCL, MCL, and DMCL.

**Conclusion:**

The intact and ACL-sectioned models were validated against cadaveric experimental results for all rotations. It is acknowledged that the validation criteria are very lenient; further refinement is required for improved validation. The results indicate that anterolateral augmentation moves the kinematics closer to the intact knee state; combined ACLR and ALLR provide the best outcome for this specimen.

**Supplementary Information:**

The online version contains supplementary material available at 10.1186/s12938-023-01094-y.

## Introduction

Despite continued advances in ACLR procedures, the failure rate is reported as high as 14% [1, 2]. Residual rotational instability is the reported reason for up to 25% of these ACLR failures [3–5]. This anterolateral instability is caused by damage to the anterolateral ligament complex at the time of ACL injury and typically presents as anterolateral rotational laxity. Techniques such as modifying ACL graft tunnel positions [6, 7] or using an anatomic double-bundle ACLR [8, 9] are not always sufficient to control this anterolateral rotational laxity [10].

An alternate approach is to combine ACLR with an anterolateral extra-articular reconstruction [11–17]. This concept pre-dates modern intra-articular reconstruction techniques, and several anterolateral extra-articular procedures have been developed [18–21], which have been shown to reduce anterolateral rotational instability [15, 22]. However, determining which method controls anterolateral rotational laxity without potentially over-constraining the joint remains challenging [23–25].

Neri et al. [25, 26] in a recent cadaveric study compared several of these anterolateral techniques. They found that adding either anterolateral ligament reconstruction or modified Ellison procedures returned the reconstructed knee closest to the native knee kinematics. A return to natural knee kinematics is thought to provide an ideal mechanical environment for the ACL graft during its integration [27]. However, they also reported that some anterolateral procedures, while improving overall kinematics, also appear to cause increased lateral tibiofemoral compartment pressures [25, 26]. Evidence suggests that this may accelerate knee osteoarthritis [28].

This study aimed to complement the cadaveric studies [25, 26] by developing a subject-specific musculoskeletal computational model based on one of the cadaveric specimens. Kinematic data for the intact and ACL-sectioned cadaveric knee were used for validation. This model was then modified to computationally assess the impact of the different reconstructions applied during the cadaveric study on the knee kinematics and ligament strains.

We hypothesised that our computational model would: accurately capture the rotations and translations of the tibiofemoral joint for passive knee flexion; approximate the strain present in the knee ligaments throughout these movements; and correlate well with the results of the previous cadaveric study [26]. Replicating these passive knee outcomes should provide an important step toward developing a computational model which can be used as a clinical tool for planning and optimising patient-specific augmented ACLR procedures.

## Results

### Model validation

**Fig. 1:**
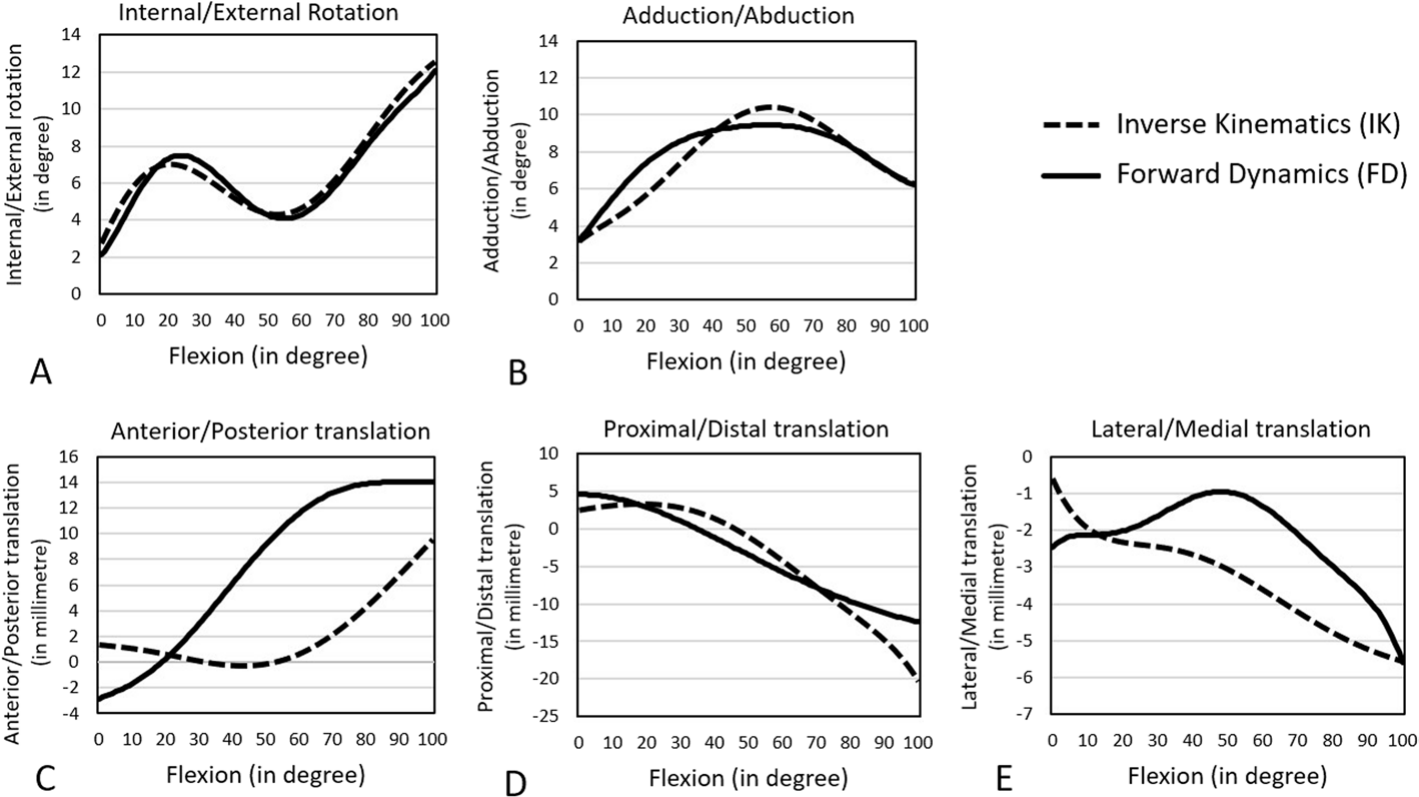
Tibiofemoral kinematics (tibia relative to the femur) of the intact model measured by FD (solid line) and IK (dashed line) during 0–100° of passive knee flexion

**Fig. 2:**
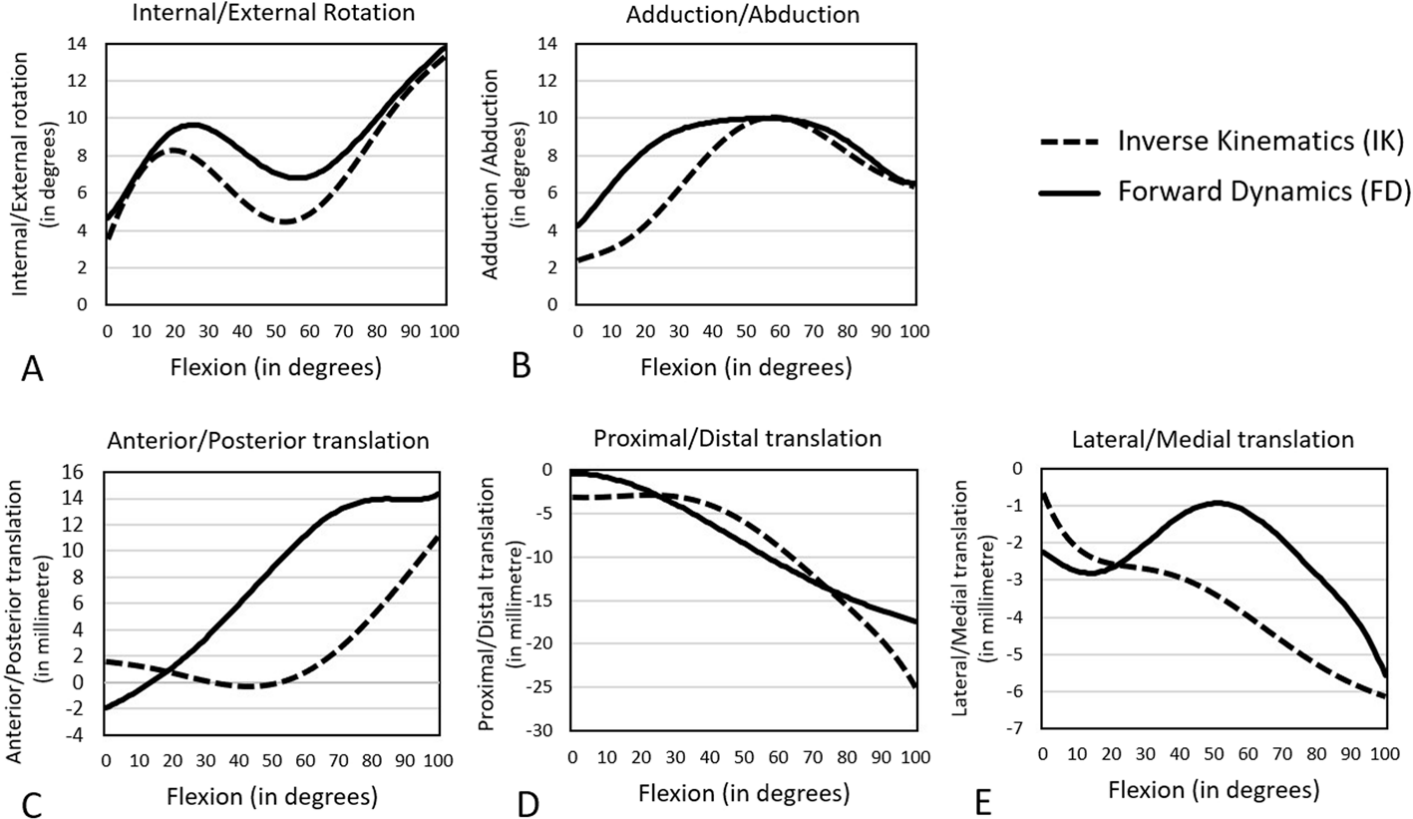
Tibiofemoral kinematics (tibia relative to the femur) of the ACL-sectioned model measured by FD (solid line) and IK (dashed line) during 0–100° of passive knee flexion

For both the intact and ACL-sectioned knees, a kinematics comparison of inverse kinematics (IK) and forward dynamics (FD) data are presented in Figs. 1, 2, and Table 1. IK and FD data reflect cadaveric [25, 26] and musculoskeletal model predicted kinematics, respectively. The FD internal rotation (IR) pattern of the intact and ACL-sectioned knee models was similar to the IK results (Figs. 1, 2). This was reflected in NRMSE values below 30% for most DoFs. The only exception was the anterior–posterior translation, which was around 75% and 60% for the intact and ACL-sectioned knees, respectively (Table 1).

**Table 1:** Comparison of tibiofemoral kinematics (tibia relative to the femur) in the intact and ACL-sectioned models between IK and FD methods during 0–100° of passive knee flexion

Orientation of motion	Range of motion		Maximum difference between IK and FD	NRMSE (%)
	IK	FD		
**Intact knee model**				
Internal/external rotation	2.7 to 12.5 (range 9.8°)	2.1 to 11.8 (range 9.7°)	0.8° at 30° of knee flexion	5.4^a^
Adduction/abduction	3.3 to 6.4 (range 3.1°)	3.4 to 6.4 (range 3°)	1.7° at 22° of knee flexion	11.8^a^
Anterior/posterior translation	1.3 to 9.3 (range 8 mm)	− 2.6 to 14 (range 16.6 mm)	6.7 mm at 100° of knee flexion	74.6
Proximal/distal translation	2.5 to − 20.3 (range 22.8 mm)	4.5 to − 12.4 (range 16.9 mm)	7.9 mm at 100° of knee flexion	10.6^a^
Lateral/medial translation	− 0.8 to − 5.7 (range 4.9 mm)	− 2.4 to − 5.6 (range 3.2 mm)	2.1 mm at 50° of knee flexion	30.4
**ACL-sectioned knee model**				
Internal/external rotation	3.9 to 13.4 (range 9.5°)	4.9 to 13.6 (range 8.7°)	2.5° at 50° of knee flexion	16.5^a^
Adduction/abduction	2.5 to 6.5 (range 4°)	4.4 to 6.5 (range 2.1°)	3.8° at 20° of knee flexion	26.7^a^
Anterior/posterior translation	1.6 to 11.1 (range 9.5 mm)	− 1.9 to 14.3 (range 16.2 mm)	10.5 mm at 63° of knee flexion	60.5
Proximal/distal translation	− 3.2 to − 24.3 (range 21.1 mm)	− 0.5 to − 17.4 (range 16.9 mm)	6.9 mm at 100° of knee flexion	11.2^a^
Lateral/medial translation	− 0.9 to − 6.2 (range 5.3 mm)	− 2.3 to − 5.6 (range 3.3 mm)	2.55 mm at 54° of knee flexion	30.7

*IK* inverse kinematics, *FD* forward dynamics, *NRMSE* normalised root mean square error

^a^Indicates an acceptable result compared to the cadaveric data (NRMSE < 30%)

**Fig. 3:**
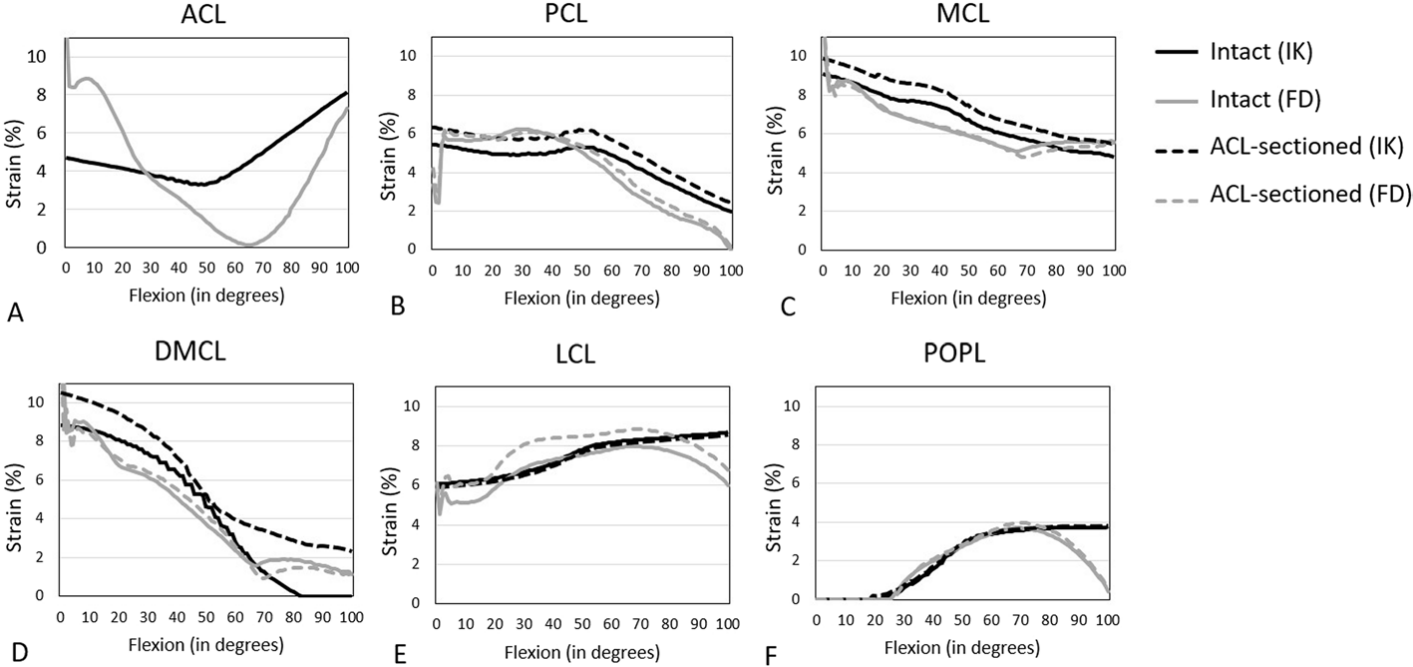
Representation of the IK and FD ligament strain values within the ACL (anterior cruciate ligament) (**A**), PCL (posterior cruciate ligament) (**B**), MCL (medial collateral ligament) (**C**), DMCL (deep medial collateral ligament) (**D**), LCL (lateral collateral ligament) (**E**), and POPL (popliteofibular ligament). **F** The results are presented for the intact (solid line) and ACL-sectioned (dashed line) knee states during passive knee flexion from 0 to 100° with applied 5Nm IR (internal rotation) torque [25, 26]

The FD ligament strains in the intact, and ACL-sectioned knees have been compared with the IK strains calculated using the experimental kinematics [26] (Fig. 3; Table 2). The FD ligament strains were within 13.9–63.8% NRMSE of the respective IK values across the entire flexion range in the intact knee. Across ligaments, NRMSE values were less than 34.6% in strains for PCL, MCL, DMCL, LCL, and POPL (Table 2) and substantially greater for ACL, with 63.8% of NRMSE in the intact knee. Considerable differences in strain profile were seen for ACL strain values in the whole flexion range. However, similarities in the patterns for the remaining ligament strain curves are evident.

**Table 2:** NRMSE between the IK and FD strains within the ACL, PCL, MCL, DMCL, LCL, and POPL ligaments during passive knee flexion from 0 to 100° with 5Nm IR (internal rotation) torque

Knee state	NRMSE ligament strain (%)					
	ACL	PCL	MCL	DMCL	LCL	POPL
Intact	63.8	34.6	20.1^a^	13.9^a^	33.6	22.1^a^
ACL-sectioned	–	32.4	30.1	21.4^a^	33.1	20.7^a^
ACL-reconstructed alone (ACLR)	63.1	32	29.9^a^	19.8^a^	35	24.7^a^
ACLR combined with anterolateral ligament reconstruction (ACLR + ALLR)	55.6	18.4^a^	23.8^a^	14.1^a^	35.1	23.2^a^
ACLR combined with MacIntosh reconstruction (ACLR + Mac)	66.5	24.1^a^	24.6^a^	16.4^a^	42.4	25.3^a^
ACLR combined with Ellison reconstruction (ACLR + Ell)	43.6	21.9^a^	27.5^a^	18.6^a^	39.8	27.2^a^
ACLR combined with deep-Lemaire reconstruction (ACLR + DL)	47.8	20.1^a^	24.6^a^	15.9^a^	42.1	26.1^a^

^a^Indicates an acceptable result compared to the cadaveric data (NRMSE < 30%)

### Evaluation of reconstructed knee model predictions

Following validation of the intact and ACL-sectioned knee models, the tibiofemoral kinematics and ligament strains of the reconstructed FD knee models were measured and compared against the experimental IK values.

### Predicted kinematics

**Fig. 4:**
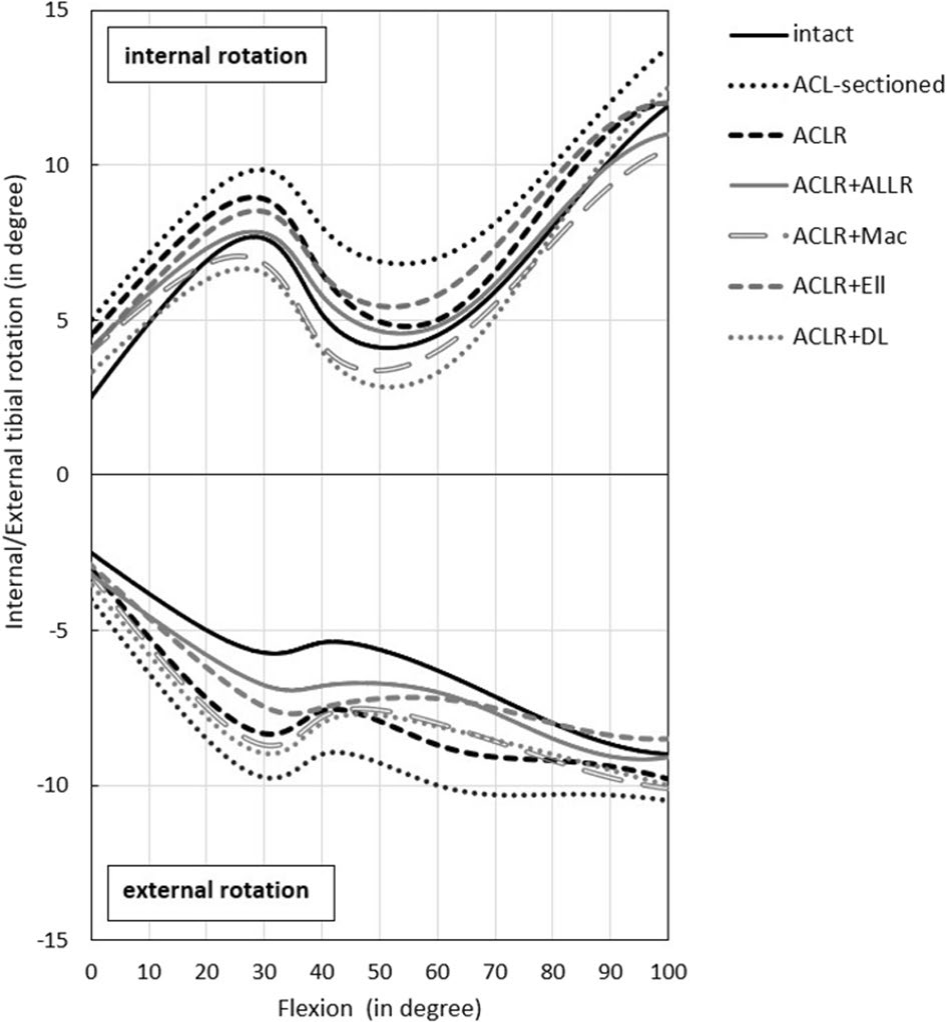
The FD kinematic response to 5Nm IR (internal rotation) and ER (external rotation) torque. The results are presented for intact, ACL-sectioned, ACLR (anterior cruciate ligament reconstruction), ACLR + ALLR (ACLR combined with the ALL-reconstructed knee), ACLR + Mac (ACLR + modified MacIntosh), ACLR + Ell (ACLR + modified Ellison) and ACLR + DL (ACLR + deep-Lemaire), models during 0–100° of passive knee flexion

The FD rotational pattern of the tibia relative to the femur (internal rotation (IR) and external rotation (ER)) across the different reconstructed knee models were predicted (Fig. 4) during the 0–100° of passive knee flexion. A comparison with IK kinematics under 5Nm IR torque [25, 26] is also presented in Table 3. The FD IR results reflect the trends in the cadaveric experiment [26], with the ACLR + ALLR procedure restoring the overall IR kinematics through the full flexion range to the intact knee. However, the FD IR values showed a smaller range of variation than the experimental results [26]. The 5Nm ER [25, 26] torque caused the models to externally rotate by around 2.5–10°, compared to the literature range of 0–30° [29, 30], across 0–100° of knee flexion.

**Table 3:** A comparison of internal tibial rotations predicted by FD models and experimentally measured results by Neri et al. [26] under 5 Nm IR (internal rotation) torque

Knee state	Internal rotation (IR) range	
	FD model (°)	Neri et al. [26] (°)
Intact	2.5 to 12 (range 9.5)	0 to 12.1 (range 12.1)
ACL-sectioned	4.9 to 13.8 (range 8.9)	2.5 to 14 (range 11.5)
ACL-reconstructed alone (ACLR)	4.4 to 12 (range 7.6)	2 to 12.2 (range 10.2)
ACLR combined with anterolateral ligament reconstruction (ACLR + ALLR)	4.1 to 11 (range 6.9)	0.5 to 12.1 (range 11.6)
ACLR combined with MacIntosh reconstruction (ACLR + Mac)	3.9 to 10.5 (range 6.6)	− 1 to 10.1 (range 11.1)
ACLR combined with Ellison reconstruction (ACLR + Ell)	3.9 to 12 (range 8.1)	0.5 to 12.8 (range 12.3)
ACLR combined with deep-Lemaire reconstruction (ACLR + DL)	3.3 to 12.5 (range 9.2)	− 1.9 to 9.8 (range 11.7)
Average of IR range (mean ± SD)	Range 8.1 ± 1.1	Range 11.5 ± 0.7

### Predicted ligament strains

**Fig. 5:**
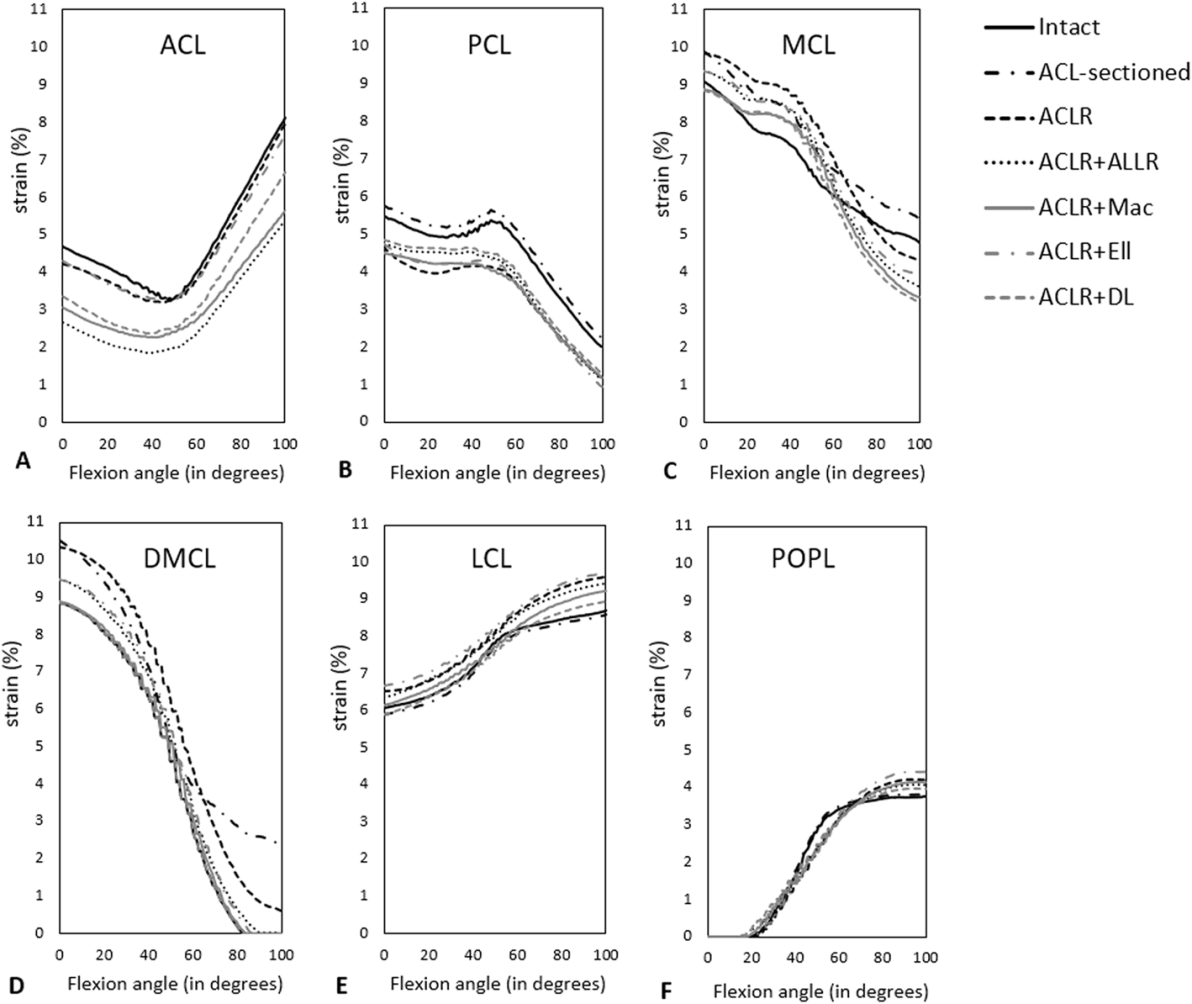
FD ligament strains, within the ACL (anterior cruciate ligament) (**A**), PCL (posterior cruciate ligament) (**B**), MCL (medial collateral ligament) (**C**), DMCL (deep medial collateral ligament) (**D**), LCL (lateral collateral ligament) (E), and POPL(popliteofibular ligament) (**F**), during passive knee flexion from 0 to 100° with applied 5Nm IR torque. The results are represented for different knee states, including the intact, ACL-sectioned, ACLR (anterior cruciate ligament reconstruction), ACLR + ALLR (ACLR combined with the ALL-reconstructed knee), ACLR + Mac (ACLR + modified MacIntosh), ACLR + Ell (ACLR + modified Ellison) and ACLR + DL (ACLR + deep-Lemaire)

The predicted FD ligament strains for the knee model with 5Nm IR applied torque [25, 26] are presented in Fig. 5. Compared with the intact model, the ACL-sectioned model showed a uniform increase in strain values across the PCL, MCL, POPL and DMCL. In contrast, the LCL experienced a uniform strain decrease in the ACL-sectioned model. The ACLR + ALLR produced the greatest overall reduction in ACL graft strain throughout the flexion cycle (Fig. 5A). ACLR + ALLR, ACLR + Mac, and ACLR + DL showed reduced ACL, MCL, DMCL, LCL, and POPL strains throughout the full flexion cycle compared to strains following isolated ACLR. The ACLR + Ell technique resulted in smaller strain values in MCL and DMCL throughout the full flexion cycle compared to isolated ACLR. In contrast, the strain patterns of ACL, PCL, LCL, and POPL changed slightly among the ACLR + Ell technique.

NRMS errors between the IK and FD strains within the ACL, PCL, MCL, DMCL, LCL and POPL bundles with 5 Nm IR applied torque [25, 26] have also been calculated and reported in Table 2. The maximum NRMS differences between the IK and FD strains within the ACL and PCL were seen in the intact knee with 63.8% and 34.6%, respectively. In comparison, the MCL (with NRMSE of 20.1%), and the DMCL (with NRMSE of 13.9%), showed the lowest NRMSE in the intact knee compared to the other knee states (Table 2). LCL and POPL had lower NRMSE in the intact (with NRMSE of 33.6% and 22.1%, respectively) and ACL-sectioned (with NRMSE of 33.1% and 20.7%, respectively) than the rest of the knee states. The NRMSEs for PCL, MCL, DMCL, LCL, and POPL ligament strains were on the order of 20–30%, while that relating to ACL was around 40–60% (Table 3).

## Discussion

This musculoskeletal modelling study developed an OpenSim model of the intact knee consisting of subject-specific tibiofemoral contact surfaces and 19 ligament bundles. The predicted FD model kinematics were validated against experimental IK results for the matching cadaveric knee in intact and ACL injured states. Following validation, the reconstructed knee model was created by modifying the intact model to reflect the reconstruction grafts. FD was used to calculate the knee kinematics and ligament strains, which were compared to the cadaveric study data to determine which procedure best restored the knee to intact kinematics. The results indicate that anterolateral augmentation moves the kinematics closer to the intact knee state; combined ACLR and ALLR provides the best outcome for this specimen.

### Model validation

The musculoskeletal model was validated for the intact and ACL injured states by comparing the predicted FD kinematics of these two models to corresponding experimental data. The ACL injured model was based on the intact model, with the ACL and ALL removed. It is acknowledged that the criterion for validation is very lenient (NRMSE < 30%). This was due to the ad hoc method of modifying the model to try to match the experimental kinematics.

The primary focus of this study was rotations, as these are typically the focus of both clinical and computational papers. However, tibiofemoral translations were also reported. FD rotational knee kinematics matched with the cadaveric experimental results for both intact and ACL-sectioned models (NRMSE < 30%) [31, 32]. The ranges of motion were also within the physiological range of motion [33–35]. Differences between the FD-predicted results and the cadaveric experimental data were more obvious in the translation results. However, the only anterior–posterior translation did not meet the acceptance criteria, with NRMSE > 30%. Differences between the FD and IK data are most likely attributed to the model's contact surfaces, ligament slack lengths, and insertion points, which should be further refined and optimised to produce a closer match [36–40].

The predicted ligament strains for the intact and ACL-sectioned models matched reasonably well with the experimental results calculated using IK in OpenSim [26]. The average NRMSE was less than 26%, except for the ACL strain in the intact case (NRMSE = 63.8%). The errors that can be seen highlight the results' sensitivity to slight variations in contact geometry and ligament placement.

The predicted ligament strain values agreed with other musculoskeletal modelling studies [41, 42], suggesting that applying a 5Nm IR torque [25, 26] increases MCL and DMCL strain at full extension and increases PCL, LCL, and POPL bundle strain in flexion. The published ACL strains are lower than those predicted by our FD model. The considerable difference in ACL strain values between the IK and FD results suggests that the model might not accurately predict the ACL strains. The authors hypothesise that perhaps the central location of the ACL makes it more sensitive to changes in contact geometry and the exclusion of other soft tissues, such as the menisci.

Finally, it is also worth noting that the maximum ligament strain values achieved during the passive flexion motion of the knee model of this study were 10% (Fig. 3) which is in close agreement with the limit proposed by Blankevoort et al. [43], adding to the validation of the defined ligament slack lengths.

### Evaluation of reconstructed knee model predictions

Predicted kinematics: The predicted IR pattern of the reconstructed knee models generally reflected the cadaveric IR values [26]. The isolated ACLR model did not restore intact knee kinematics, consistent with the experimental results [26], supporting previous findings that the isolated ACLR can lead to residual rotational instability [4, 5].

Among the various anterolateral procedures investigated, this modelling study revealed that the ACLR + ALLR better restored the overall IR kinematics through the full flexion range toward the intact state, which correlated well with the experimental studies [25, 26, 44]. Neri et al. [25, 26], in a biomechanical study of 10 cadaveric knees that underwent ACLR combined with various lateral reconstructions, demonstrated that the ACLR + ALLR procedure provided additional rotational control whilst protecting the ACL graft without risking over-constraint of the joint. Sahanand et al. [44], in a clinical study of 25 patients who underwent ACLR + ALLR with an average of 31.5 months follow-up, reported a significant improvement in the patient outcomes compared to pre-surgery, with no graft failure and residual instability.

This modelling study showed that the ACLR + Ell procedure also provided rotational control whilst protecting the ACL graft without risking over-constraint for 0–40˚ of flexion cycle, compared to the isolated ACLR knee (Fig. 4). Our results confirm the findings of Neri et al. [26], who reported that the ACLR + Ell procedure could restore physiological kinematics. Also, our results support Devitt et al. [45], who suggested that ACLR + Ell can reduce anterolateral instability in the anterolateral capsule-injured knee and restore kinematics close to the intact state. While the ACLR + Mac and ACLR + DL models also provided rotational control for IR (Fig. 4), they over-constrained the knee kinematics throughout the flexion range (Fig. 5). These findings are supported by Neri et al. [25, 26], whose cadaveric results indicated the possibility of over-constraining the joint using these procedures. Also, Geeslin et al. [46] reported that the combination of ACLR + DL procedure resulted in both over-constraint of the joint and significant reductions in tibiofemoral motion at most knee flexion angles.

As defined by applying the 5 Nm internal and external torques [25, 26], the limits of tibial rotation are shown in Fig. 4 as a flexion function for all the knee models. These limits determine the freedom-of-motion range of the developed knee model, from full extension up to 100° of flexion, and are considered to comprise the rotation envelope of passive knee motion. Overall, the rotation envelope of passive knee flexion (IR and ER) presented in this developed model (Fig. 4) compares well with our experimental data (Table 3) [26] and similar literature [29, 30, 47–49] further supporting the validity of our knee models.

Predicted ligament strains: This study estimated the strains in the major knee ligaments based on musculoskeletal modelling techniques (Fig. 5). These findings indicate that ACLR combined with anterolateral procedures reduced the strain levels within the ACL graft compared to the isolated ACLR technique. Specifically, the ACL + ALLR technique had the greatest reduction in ACL graft strains, which supports evidence that this technique improves knee kinematics immediately post-surgery and may improve patient outcomes in the longer term [26, 50]. Specifically, all of the anterolateral augmented ACLR techniques saw a reduction of strain in the ACL graft to levels typical of a healthy knee, which is likely to be beneficial in protecting the ACL graft during healing, and in doing so, leading to improved outcomes [49].

Figure 4 shows that ACLR + DL and ACLR + Mac result in over-constrained internal tibia rotation compared to the other lateral extra-articular methods. In all the models, the axial rotation was restrained mainly by the MCL and DMCL bundles (with a 0–10.5% strain range) and less so with LCL and POPL (Fig. 5 and Table 2), which also agrees with the literature on ligament function [51–53].

This study also showed that as the ACL-sectioned knee model was passively flexed with 5Nm of IR torque [25, 26], LCL experienced a uniform reduction in strain values than the intact knee (Fig. 5E). These results are different from current thinking on LCL ligament elongation behaviour reported in the literature [54–56] and can likely be attributed to differences in the location of ligament attachment sites or ligament slack length values in the model.

## Limitations

The main limitation of this model is related to the manual, ad hoc method of generating the contact surfaces. This method removed the possibility of achieving a close match between the cadaveric and predicted results and led to the adoption of a more lenient validation criteria for the musculoskeletal model. Further, the manual method of extracting the cartilage geometries means these results are not easily reproducible. Future work should develop a reproducible computational process for generating these contact surfaces and driving them towards an optimum which fulfils more stringent validation criteria. Developing an appropriate computational optimisation program was beyond the scope of this study. However, the agreement achieved between experimental and predicted results using our methodology highlights the potential for achieving a fully validated model in future studies. In addition, while the resulting surfaces created a reasonable approximation of the patient geometry, it must be acknowledged that critical soft tissue, such as the menisci, was not included. While there is precedent for this in several seminal papers on musculoskeletal modelling [36, 57–59], this omission has likely contributed to some of the higher NRMSE values found in this study. The menisci play an important role in stabilising the joint, providing cushioning, and restricting excessive movement by ensuring the femoral condyles are guided through motion [60, 61]. Also, it is noted that the torque applied during the experiment [26] was not continuously monitored. As the model applied a constant torque, any deviations from this during the experiment would lead to differences in the kinematics and strain outputs. Finally, the differences observed between our predicted FD knee kinematics and the experimental results [26] may be due to this study using a subject-specific knee model based on only one of the ten cadaveric models used in the experiment.

## Conclusion

To our knowledge, this is the first study to provide a direct comparison between knee joint kinematics and ligament strains predicted by an OpenSim FD model and those measured experimentally for ACL reconstruction with different lateral augmentation procedures. The ability to approach validation of our musculoskeletal knee model by correlating it against an accepted cadaveric model is a crucial step in creating a non-invasive computational tool for assessing and optimising ACLR procedures for individual patients. A comparison of various currently used anterolateral augmentation procedures revealed that adding those procedures to the isolated ACLR restored intact knee kinematics in an ACL-sectioned knee. In comparison, combined ACLR and ALLR provide the best outcome for this specimen.

## Materials and methods

### Experimental data

This study utilised data from a previous controlled laboratory experiment that assessed the knee kinematics and lateral compartment pressures of ten lower extremities cadaveric specimens [25, 26]. For all knee states, knee kinematics and contact pressures were acquired for three cycles of passive knee flexion/extension combined with 5 Nm of internal rotation (IR) torque [25, 26] applied manually to the tibia. The test was repeated with 5 Nm of external rotation (ER) [25, 26]. To track the knee kinematics, the limb was prepared with four bi-cortical pins (two for the femur and two for the tibia) with two retro-reflective markers fixed to each pin using a validated protocol [62]. Knee kinematics were acquired using a 3D optoelectronic motion capturing system consisting of five Bonita cameras on tripods (Vicon, LA, USA). A test with neutral rotation was also conducted, but these results were excluded from this study because the lack of applied torque led to an excessive amount of variation between flexion cycles due to knee laxity.

### Knee model development

**Fig. 6:**
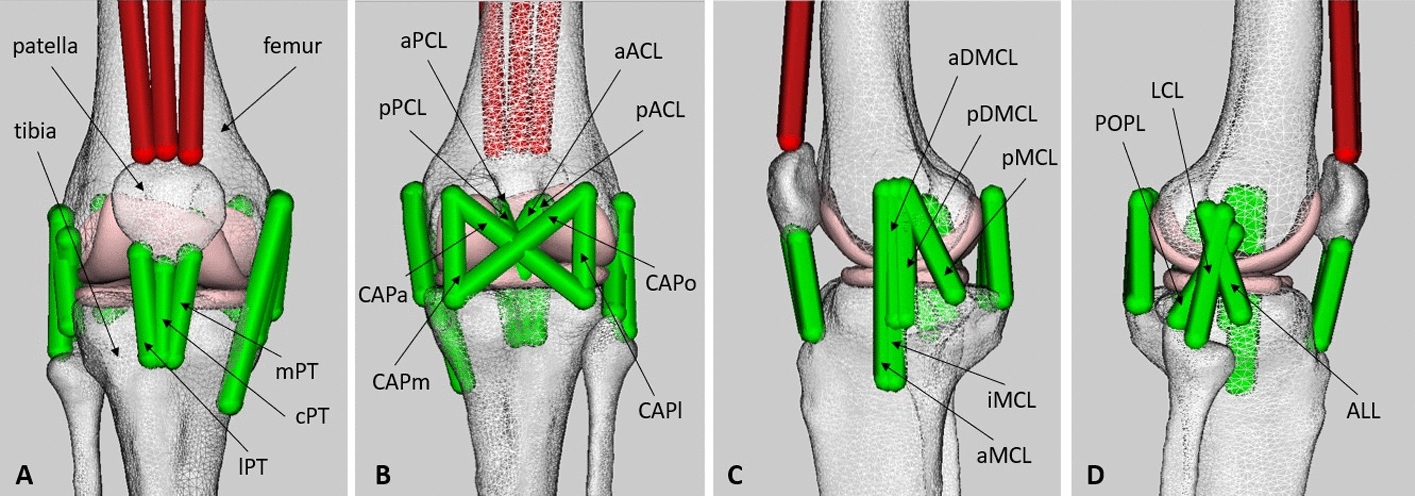
Subject-specific intact knee model developed in OpenSim [63] including one six-DoF tibiofemoral joint, one three-DoF patellofemoral joint, ligaments, and capsular bundles. Anterior view (**A**), posterior view (**B**), medial view (**C**), and lateral view (**D**). aACL and pACL: anterior cruciate ligament (anterior and posterior bundle); aPCL and pPCL: posterior cruciate ligament (anterior and posterior bundle); aMCL, iMCL and pMCL: medial collateral ligament (anterior, intermediate and posterior superficial bundle); aDMCL and pDMCL: deep medial collateral ligament (anterior and posterior deep bundle); LCL: lateral collateral ligament; POPL: popliteofibular ligament; ALL: anterolateral ligament; CAPa, CAPo, CAPm and CAPl: posterior capsule (anterior, oblique, medial, and lateral bundle); cPT, mPT and lPT: patellar tendon (central, medial and lateral bundle)

An intact, subject-specific musculoskeletal knee model consisting of a six-DoF tibiofemoral joint, a three-DoF patellofemoral joint, ligaments and capsular bundles was created in OpenSim (R3.3, Simtk, USA) [63] (Fig. 6). The intact knee model was initially adapted from Xu et al.'s [64] and Schmitz et al.'s [41] models and then further developed by incorporating subject-specific contact surfaces (Fig. 6).

Three-dimensional specimen-specific bone and cartilage geometries were reconstructed through the segmentation of CT and MR images. The contact surfaces were further amended using Fusion 360 software (R2020, Autodesk, USA) to guarantee medial and lateral contact over the entire natural knee rotation and match the experimentally measured kinematics for the intact and ACL-sectioned models [26]. Joint centres and bony landmarks, along with the precise location of drilled holes in the femur and tibia for reconstruction surgery, were also determined from segmented CT images (see Sects. 1.1–1.3 in Additional file 1).

Nineteen ligament bundles were included in the intact knee model (Table 4), with attachment points obtained from the CT and MRI images and the literature [65–74] (Fig. 6; Table 4). Two bundles represented the anterior cruciate ligament (ACL): anterior and posterior bundles (aACL, pACL). The posterior cruciate ligament (PCL) was represented by two bundles; anterior and posterior bundles of the posterior cruciate ligament (aPCL, pPCL). The medial collateral ligament (MCL) was divided into two groups of ligament bundles: superficial and deep. The superficial layer of MCL was further divided into three bundles: anterior, inferior, and posterior (aMCL, iMCL, pMCL). The deep layer of MCL was subdivided into anterior and posterior bundles (aDMCL, pDMCL). The lateral collateral ligament (LCL), the popliteofibular ligament (POPL), and the anterolateral ligament (ALL), were each represented with one bundle. The posterior capsule was represented by four bundles: the anterior, oblique, medial, and lateral bundles (CAPa, CAPo, CAPm, CAPl). The patellar tendon was modelled as three bundles: the central, medial, and lateral bundles (cPT, mPT, lPT).

**Table 4:** Values of normalised stiffness (*K*), zero-load length (*L*_0_), and reference strain (*ε*_r_) assumed in the model for each ligament bundle

Ligament	$K (\mathrm{N})$	${L}_{0}$(mm)	${\varepsilon }_{r}$
aACL	3600	32	0.03
pACL	4000	34	0.03
aPCL	4000	34	− 0.05
pPCL	1600	32	− 0.06
aMCL	2000	85	0.02
iMCL	2000	85	0.03
pMCL	4000	56	0.05
aDMCL	2000	58	0.02
pDMCL	1800	57	0.05
LCL	3400	49	0.05
POPL	1900	45	− 0.05
ALL	2700	43	0.05
CAPa	1350	45	0.05
CAPo	1500	43	0.05
CAPm	2000	28	0.05
CAPl	2000	27	0.05
cPT	6000	48	0.01
mPT	6000	47	0.01
lPT	6000	45	0.01

aACL and pACL: anterior cruciate ligament (anterior and posterior bundle); aPCL and pPCL: posterior cruciate ligament (anterior and posterior bundle); aMCL, iMCL and pMCL: medial collateral ligament (anterior, intermediate and posterior superficial bundle); aDMCL and pDMCL: deep medial collateral ligament (anterior and posterior deep bundle); LCL: lateral collateral ligament; POPL: popliteofibular ligament; ALL, anterolateral ligament; CAPa, CAPo, CAPm and CAPl: posterior capsule (anterior, oblique, medial, and lateral bundle); cPT, mPT and lPT: patellar tendon (central, medial and lateral bundle)

Ligament bundles were modelled as non-linear elastic springs with linear damping [47, 70, 75–77], with predefined stiffness and slack length values (Table 4). To avoid penetration of the ligament bundles into the bones, wrapping surfaces were also included in the model [41] (Sect. 1.4 in Additional file 1).

### Knee models

**Fig. 7:**
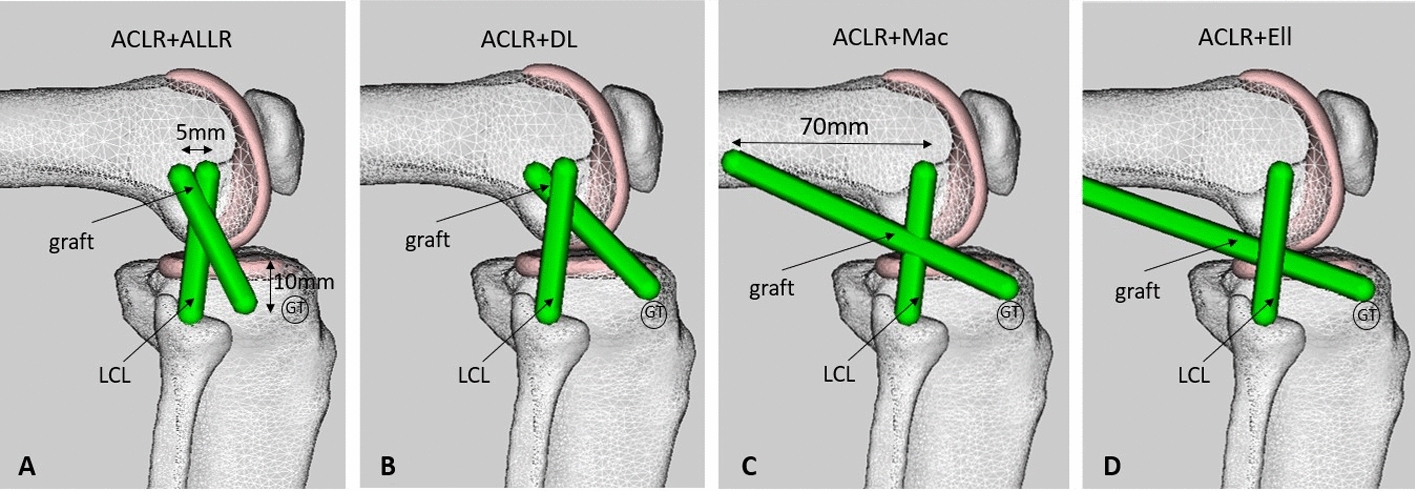
Representation of the reconstructed knee models in OpenSim [63]. ACLR combined with the ALL-reconstructed knee (ACLR + ALLR) (**A**), ACLR + deep-Lemaire (ACLR + DL) (**B**), ACLR + modified MacIntosh (ACLR + Mac) (**C**), and ACLR + modified Ellison (ACLR + Ell) (**D**). LCL: lateral collateral ligament, GT: Gerdy's tubercle

The intact OpenSim model was developed first using the process outlined above. The ACL-sectioned model was then created by removing the ACL ligament bundles from the intact model. Matching the kinematics of these two models to the corresponding cadaveric kinematic results provided validation for the OpenSim modelling process [26]. Following validation, new models (Fig. 7) were created for each reconstruction [26] by modifying the validated intact knee model, which was always used as the base model for consistency [26]. Seven of the eight knee groups explored in the Neri et al.'s cadaveric experiment [26] were modelled in this study (Fig. 7). Superficial Lemaire was excluded from the modelling as it required significant changes to the wrapping surface definitions of the validated base model used for all reconstruction models. Below are detailed descriptions of the seven modelled knee groups:

1. The intact knee.
2. The ACL-sectioned knee, in which the anterolateral ligament (ALL) was also sectioned to match the cadaveric experiment [26].
3. ACLR, in which the ACL graft was modelled by one single ligament bundle fixed with the knee at 30° and with an 80 N tension [26]. The properties of a quadrupled hamstring autograft (semi-tendinosis) [78, 79] were applied to the graft as this was the graft used in the experiment [26].
4. ACLR combined with the ALL-reconstructed knee (ACLR + ALLR) (Fig. 7A), in which the ALL graft was modelled as a single bundle ligament with properties similar to a typical gracilis graft [78, 79] passing under the ITB and over the LCL [80]. The tibial insertion point of the ALL graft was located equidistant between the centre of Gerdy's tubercle (GT) and the anterior margin of the fibular head and 10 mm distal to the joint line [81, 82]. The femoral insertion point was located 5 mm proximal and posterior to the LCL's femoral insertion [81–83].
5. ACLR + DL was similar to the ACLR + ALLR, except that the tibial tunnel of the anterolateral reconstruction was located at the centre of Gerdy's tubercle (GT) (Fig. 7B).
6. ACLR + Mac, in which the graft extended from the same tibial insertion point (GT) to the femoral insertion point located 70 mm proximal to the femoral epicondyle (Fig. 7C).
7. ACLR + Ell technique, in which the graft was modelled as a strip of the iliotibial band (ITB) detached distally, passed underneath the LCL, and finally attached to the same tibial insertion site (GT) (Fig. 7D).

In all of the reconstructed models, the lateral graft was always fixed in the same condition: in neutral rotation at 30° of flexion and with 20 Nm of applied tension characterised by a non-linear tension bundle with an average stiffness as reported in the literature [78, 79].

### Musculoskeletal simulation

The simulations were performed via OpenSim (R3.3, Simtk, USA) [63] using two main toolboxes: inverse kinematics (IK) to compute the knee joint kinematics and ligament strains from the cadaveric experiments; and forward dynamics (FD) to predict the knee kinematics and the ligament strains within the modelled knees. The pelvis and femur were fixed in all directions during the entire simulation for all simulations. For the remainder of the article, IK results will be referred to as experimental results and FD as predicted results.

For IK, the experimental marker trajectories were used to drive the tibiofemoral joint. For FD, only the flexion component of the tibia’s kinematics was prescribed, with other rotations and translations restricted only by the ligaments and contact surfaces. The FD analysis tool was used to predict the knee joint behaviour throughout the entire 100° flexion cycle while applying 5N of IR/ER torque to the tibia for all knee models [25, 26].

The FD kinematics for the intact and ACL-sectioned knee states were compared to their corresponding IK kinematics [25] to validate the OpenSim model. The kinematics measurements considered in the validation were: (1) transverse plane motion, including internal–external rotation; (2) sagittal plane motions, including anterior–posterior and proximal–distal translations; and (3) frontal plane motions, including lateral–medial translation and adduction–abduction rotation. In all three planes, the kinematics were defined as the motion of the tibia relative to the femur.

Once the intact and ACL-sectioned knee models were validated, then FD was used to predict the kinematics for all the reconstructed knee models. IR and ER torques of 5 Nm [25, 26] were applied to all the models during 0–100° of passive knee flexion. These predicted kinematics were compared to the average kinematics reported by Neri et al. [26].

The ligament strains were also compared for the cadaveric and modelled knees. For the experimental results, strains for ACL, PCL, MCL, DMCL, LCL, and POPL ligament bundles were output as part of the IK process over the full flexion range. The corresponding predicted ligament strains for the FD analysis were also output for all models. As the kinematics are validated, it is assumed that comparing the corresponding ligament strains is a valid approach.

### Data analysis

For validation, the FD model outputs of a single cadaveric specimen were compared to the IK results for that same specimen. The model outputs were discretised using MATLAB into 1° increments for easier pointwise comparison.

Since the number of specimens involved in the validation process was *n* = 1, a normalised root mean square error (NRMSE) criterion was used to compare FD results with corresponding IK results to address our hypothesis that the model accurately represents passive knee motions and ligament strains. RMSE was normalised by the range value observed for each variable. NRMSE is given by the following equation [32]:

1 $$\mathrm{NRMSE}=\frac{1}{\mathrm{max}\left(o\right)-\mathrm{min}(o) }\sqrt[2]{\frac{{\sum }_{i=1}^{N}{({p}_{i}-{o}_{i})}^{2}}{N},}$$

where $o$ is the observed value of the parameter, $p$ is the predicted value according to the model, and *N* is the total number of observations in the validation dataset.

Commonly, an NRMSE of less than 10% is considered acceptable [31, 32]. However, this criterion can vary depending on the application [84]. In this study, cadaveric data are compared with a computational model very sensitive to various modelling parameters, i.e. contact surfaces, ligament material properties, slack lengths, and insertion points. As these parameters are determined in an ad hoc manner in this study, we consider NRMSE values < 0.3 (30%) to indicate acceptable performance [85, 86]. Adopting this lenient criterion suggests the potential for a full validation under stricter criteria in future studies which will use computational methods to drive these sensitive parameters towards an optimal solution. This was beyond the scope of this study.

The ranges of FD tibiofemoral kinematics and ligament strains of our musculoskeletal models were compared to their corresponding IK results to assess the remaining reconstructed knee model outcomes. The internal–external rotation pattern of the tibia relative to the femur under 5 Nm of IR/ER torque [25, 26] across the 0–100° of passive knee flexion was measured. The strain borne by knee ligaments (ACL, PCL, MCL, DMCL, LCL, and POPL) during passive knee flexion from 0 to 100° with applied 5 Nm IR torque [25, 26] was also measured. NRMSE values were then calculated.

## Supplementary Information

**Additional file 1: Figure S1**: Subject-specific intact knee model created in OpenSim [5], including one 6-DoF tibiofemoral joint and one 3-DoF patellofemoral joint. **Figure S2**: Representation of tibiofemoral contact surfaces through different developed tibial contact geometries (the tibial plateau): planar objects (A), curvature objects (B), and subject-specific objects(C). **Figure S3**: Representation of steps developing the subject-specific tibial contact surface (the tibial plateau), including, Boolean subtraction (A), meshing the 3D object (B), and cropping/smoothing (C). **Figure S4**: Representation of three wrapping objects included in the knee model placed at the medial epicondyle, lateral epicondyle, and patellofemoral joint. **Table S1**. Wrapping object parameters.

## Data Availability

The datasets generated and analysed during the current study are available from the corresponding author on reasonable request.

## Abbreviations

ACL: Anterior cruciate ligament
ALL: Anterolateral ligament
ACLR: Anterior cruciate ligament reconstruction
ACLR + ALLR: ACLR combined with the ALL-reconstructed knee
ACLR + Mac: ACLR combined with modified MacIntosh
ACLR + Ell: ACLR combined with modified Ellison
ACLR + DL: ACLR combined with deep-Lemaire
NRMSE: Normalised root mean square error
RMSE: Root mean square error
IK: Inverse kinematics
FD: Forward dynamics
IR: Internal rotation
ER: External rotation
DoF: Degrees of freedom
aACL: Anterior bundle of anterior cruciate ligament
pACL: Posterior bundle of anterior cruciate ligament
PCL: Posterior cruciate ligament
aPCL: Anterior bundle of posterior cruciate ligament
pPCL: Posterior bundle of posterior cruciate ligament
MCL: Medial collateral ligament
aMCL: Anterior bundle of medial collateral ligament
iMCL: Inferior bundle of medial collateral ligament
pMCL: Posterior bundle of medial collateral ligament
DMCL: Deep medial collateral ligament
aDMCL: Anterior bundle of deep medial collateral ligament
pDMCL: Posterior bundle of deep medial collateral ligament
LCL: Lateral collateral ligament
POPL: Popliteofibular ligament
CAP: Posterior capsule
CAPa: Anterior bundle of posterior capsule
CAPo: Oblique bundle of posterior capsule
CAPm: Medial bundle of posterior capsule
CAPl: Lateral bundle of posterior capsule
PT: Patellar tendon
cPT: Central bundle of patellar tendon
mPT: Medial bundle of patellar tendon
lPT: Lateral bundle of patellar tendon
ITB: Iliotibial band
GT: Gerdy's tubercle
