# Supplementary material for: Towards a validated musculoskeletal knee model to estimate tibiofemoral kinematics and ligament strains: comparison of different anterolateral augmentation procedures combined with isolated ACL reconstructions
Source: Biomed Eng Online. 2023 Mar 27;22:31. doi: 10.1186/s12938-023-01094-y (PMC10044816; doi:10.1186/s12938-023-01094-y)
Supplement: Supplementary file 1 — Additional file 1: Figure S1: Subject-specific intact knee model created in OpenSim [5], including one 6-DoF tibiofemoral joint and one 3-DoF patellofemoral joint. Figure S2: Representation of tibiofemoral contact surfaces through different developed tibial contact geometries (the tibial plateau): planar objects (A), curvature objects (B), and subject-specific objects(C). Figure S3: Representation of steps developing the subject-specific tibial contact surface (the tibial plateau), including, Boolean subtraction (A), meshing the 3D object (B), and cropping/smoothing (C). Figure S4: Representation of three wrapping objects included in the knee model placed at the medial epicondyle, lateral epicondyle, and patellofemoral joint. Table S1. Wrapping object parameters. [file 12938_2023_1094_MOESM1_ESM.docx]

Additional file 1

# Modelling Of Articulating Geometries

## Model coordinate systems

The origin of the femur coordinate system was placed at the centre of the femoral head with the x-axis pointing anteriorly, the y-axis superiorly, and the z-axis to the right [1, 2]. The y-axis was oriented along a line connecting the femoral head and the centre of the femoral condyles. The tibial coordinate system was located at the midpoint of the femoral condyles with the knee in a full extension based on the measurements taken from CT reference positions. The coordinate system for the patella was placed at the distal pole of the patella. The tibial and patella coordinates orientation was the same as the femur coordinate system when in the reference position (Figure S1)[1, 2].

The tibiofemoral joint kinematics represents the movement of the tibia relative to the femur. The tibiofemoral joint was modelled as a six-DoF joint, including three rotational DoFs and three translational DoFs [3]. Abduction-adduction, internal-external rotations, and knee flexion-extension are the rotational DoFs about X-axis, Y-axis, and Z-axis (Figure 1). The translational DoFs consist of anterior-posterior translations, proximal-distal translations, and medial-lateral translations along the X-axis, Y-axis, and Z-axis.

The patellofemoral joint is modelled with 3 DoF. Only one DoF, the translation of the patella along the Y‐axis of the femur, is free. The other two-DoFs, the translation along the X‐axis and rotation about the Z‐axis, were prescribed Y-axis translation functions (Figure S1) [3, 4].


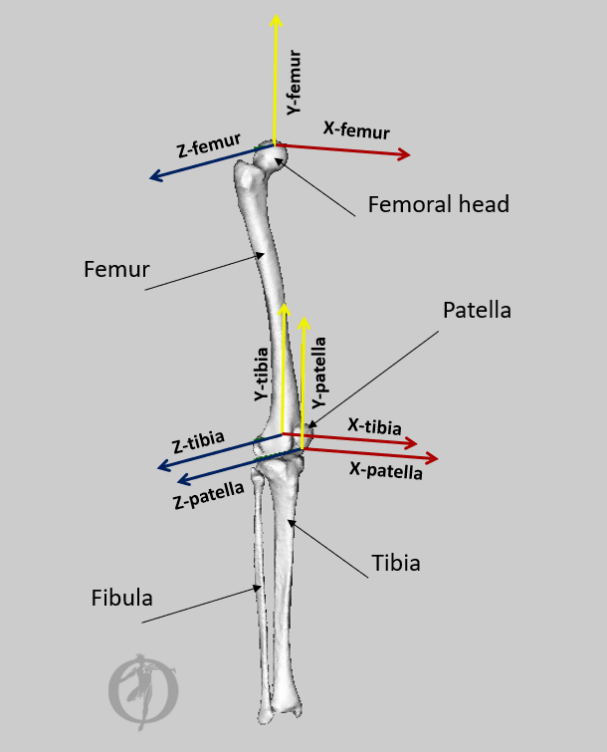


Figure S1: Subject-specific intact knee model created in OpenSim [5], including one 6-DoF tibiofemoral joint and one 3-DoF patellofemoral joint.

## Imaging and segmentation

3D specimen-specific bone and femoral cartilage geometries were created through semi-automatic CT and MR images segmentation using Amira 6.7.0 (Thermo Fisher Scientific, USA) and exported to Geomagic Studio 2012.1.0 (Geomagic, USA) software.

To extract the intact knee geometry, including the bones and femoral cartilage along with connective tissue bundles representing ligaments and capsules, the cadaveric lower body underwent a pre-operative high-resolution MRI (3T, Magnetom Skyra; Siemens AG Healthcare) with a 15-channel phased-array send/receive knee coil (Siemens AG Healthcare) and CT (Aquilion PRIME; Toshiba Medical Systems Corporation) under protocols of the Sydney Orthopaedic Research Institute (SORI) [6]. Also, after knee lateral augmentation reconstructions, a postoperative CT was done using the same protocol to get the exact position of grafts in the femur and tibia. While imaging, the knee was fixed in full extension and neutral rotation. This position was defined as the reference position of the joint in the model. The whole leg CT data has been scanned in 1mm intervals, resulting in more than 2000 total slices (1000 slices each of the femur and tibia).

Ligament attachment positions, joint centres and bony landmarks were determined from segmented CT and MRI images using level thresholding in Amira software. Also, the precise placement of drilled holes in the femur and tibia after reconstruction surgery was identified via the segmentation of CT images. In Amira software, 3D surface models were then generated, smoothed, and exported to Geomagic Studio for further processing.

In Geomagic Studio software, the average of a three-dimensional point cloud of ligaments footprints (Cruciate and Collateral ligaments) was found using imported bone and ligament geometries. Geomagic was also used to convert the 3D geometry file format to an appropriate format for 3D musculoskeletal modelling in OpenSim [5].

## Tibiofemoral contact

Using the similar process as outlined above, the articular surfaces of the femoral cartilage was obtained from segmentation of CT and MRI images in Amira and Geomagic Studio software. Initially, the tibial plateaus were approximated with two planar surfaces adapted from Schmitz et al. [4] in OpenSim (Figure S2A). Due to the concave and convex nature of the medial and lateral tibia plateaus, tibial contact surfaces were then modelled with two curved planes (Figure S2B).


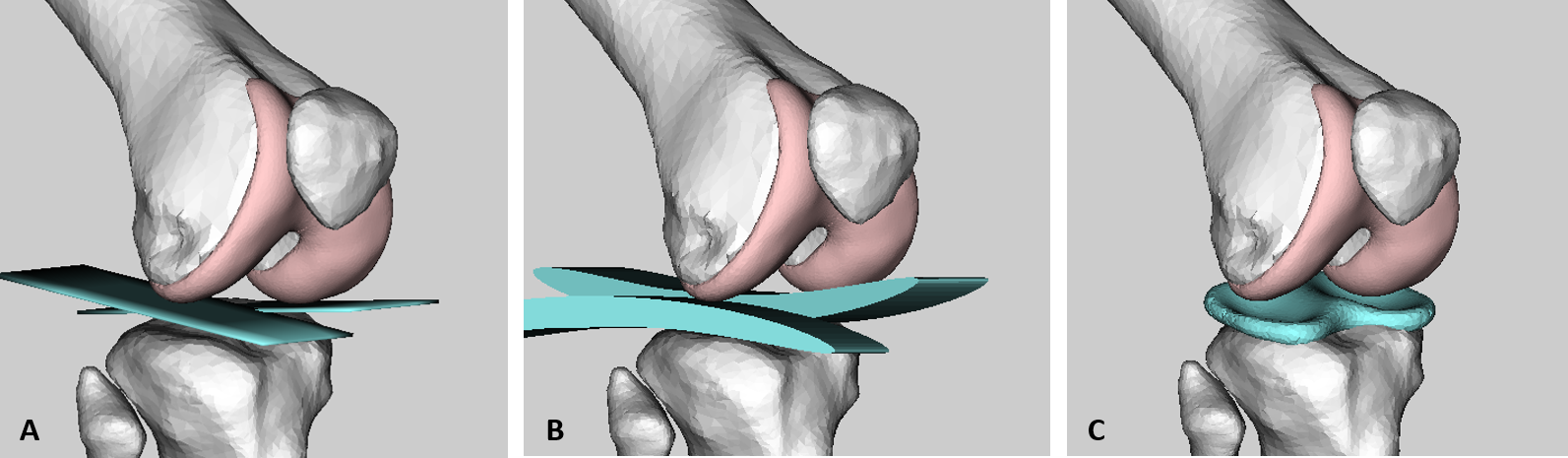


Figure S2: Representation of tibiofemoral contact surfaces through different developed tibial contact geometries (the tibial plateau): planar objects (A), curvature objects (B), and subject-specific objects(C)

To get the exact subject-specific articulating surfaces in Fusion 360 (R2020, Autodesk, USA), Boolean subtraction was used to extract the precise profile of articulating surfaces between the femoral and tibial cartilages and to guarantee both the medial and lateral contact over the entire natural knee motion. The surfaces were then smoothed using two filtering features (erase/fill and smooth) available in Autodesk Fusion 360, which flattened the spikes on the surface mesh (Figure S3B and C).

Next, the surface meshes of femoral and tibial cartilages were converted to solid geometries (.obj format) using Autodesk Fusion360. Finally, the solid geometries were imported into OpenSim (Figure S2C), where the knee model was developed. The cartilage surfaces were first configured at full knee extension based on reference data determined from CT images. At the same time, the tibia was abducted and rotated 0.0 degrees, then tuned slightly to match the kinematic results of the cadaveric experiment.


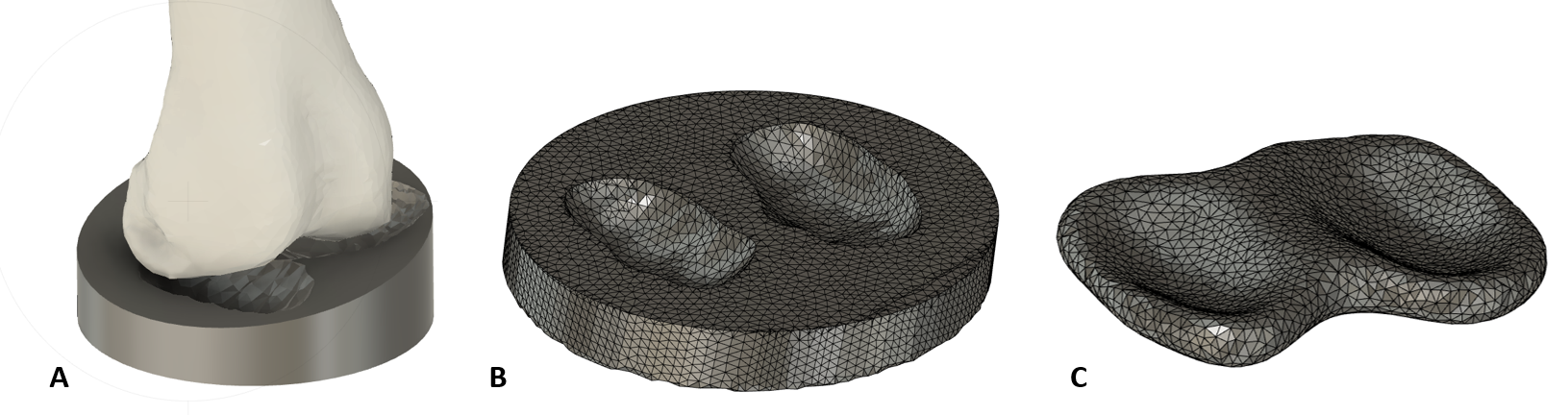


Figure S3: Representation of steps developing the subject-specific tibial contact surface (the tibial plateau), including, Boolean subtraction (A), meshing the 3D object (B), and cropping/smoothing (C)

## Wrapping objects

To avoid penetration of the ligament bundles into the bones, wrapping surfaces were also included in the OpenSim model: two spheres placed around the femoral epicondyles and one cylinder behind the patella bone (Figure S4: Table S2 ).


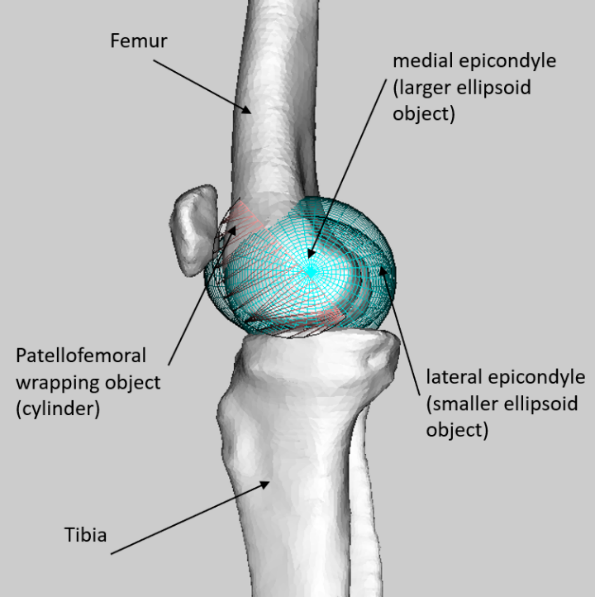


Figure S4: Representation of three wrapping objects included in the knee model placed at the medial epicondyle, lateral epicondyle, and patellofemoral joint

Table S1. Wrapping object parameters

| Wrapping object | Geometry (dimensions in meter) | Location in the femur (in meter) |
| --- | --- | --- |
| Medial epicondyle | Ellipsoid with (0.037 0.035 0.035) | (-0.005 -0.45 -0.01) |
| Lateral epicondyle | Ellipsoid with dimension (0.041 0.028 0.035) | (-0 -0.45 0.0005) |
| Patellofemoral | Cylinder with radius 0.036 | (0.007 -0.44 0) |

# References

1. Delp SL, Loan JP, Hoy MG, Zajac FE, Topp EL, Rosen JM. An interactive graphics-based model of the lower extremity to study orthopaedic surgical procedures. IEEE Trans Biomed Eng. 1990;37(8):757-67.

2. Arnold AS, Asakawa DJ, Delp SL. Do the hamstrings and adductors contribute to excessive internal rotation of the hip in persons with cerebral palsy? Gait & posture. 2000;11(3):181-90.

3. Arnold EM, Ward SR, Lieber RL, Delp SL. A model of the lower limb for analysis of human movement. Ann Biomed Eng. 2010;38(2):269-79.

4. Schmitz A, Piovesan D. Development of an Open-Source, Discrete Element Knee Model. IEEE Trans Biomed Eng. 2016;63(10):2056-67.

5. Delp SL, Anderson FC, Arnold AS, Loan P, Habib A, John CT, et al. OpenSim: open-source software to create and analyze dynamic simulations of movement. IEEE Trans Biomed Eng. 2007;54(11):1940-50.

6. Grasso S, Linklater J, Li Q, Parker DA. Validation of an MRI protocol for routine quantitative assessment of tunnel position in anterior cruciate ligament reconstruction. The American journal of sports medicine. 2018;46(7):1624-31.

7. Blankevoort L, Huiskes R. Ligament-bone interaction in a three-dimensional model of the knee. Journal of biomechanical engineering. 1991;113(3):263-9.

8. Blankevoort L, Kuiper J, Huiskes R, Grootenboer H. Articular contact in a three-dimensional model of the knee. Journal of biomechanics. 1991;24(11):1019-31.

9. Shelburne KB, Kim HJ, Sterett WI, Pandy MG. Effect of posterior tibial slope on knee biomechanics during functional activity. Journal of Orthopaedic Research. 2011;29(2):223-31.

10. Shelburne KB, Torry MR, Pandy MG. Contributions of muscles, ligaments, and the ground‐reaction force to tibiofemoral joint loading during normal gait. Journal of orthopaedic research. 2006;24(10):1983-90.

11. Wismans J, Veldpaus F, Janssen J, Huson A, Struben P. A three-dimensional mathematical model of the knee-joint. Journal of biomechanics. 1980;13(8):677-85.

12. Blankevoort L, Huiskes R, de Lange A. Recruitment of knee joint ligaments. J Biomech Eng. 1991;113(1):94-103.

13. Xu H, Bloswick D, Merryweather A. An improved OpenSim gait model with multiple degrees of freedom knee joint and knee ligaments. Comput Methods Biomech Biomed Engin. 2015;18(11):1217-24.

14. Neri T, Dabirrahmani D, Beach A, Putnis S, Oshima T, Cadman J, et al. A biomechanical comparison of the main anterolateral procedures used in combination with anterior cruciate ligament reconstruction. Orthopaedic Journal of Sports Medicine. 2020;8(2_suppl):2325967120S00002.
